# Antioxidant Activity Evaluation and Assessment of the Binding Affinity to HSA of a New Catechol Hydrazinyl-Thiazole Derivative

**DOI:** 10.3390/antiox11071245

**Published:** 2022-06-24

**Authors:** Mihaela Mic, Adrian Pîrnău, Călin G. Floare, Raluca Borlan, Monica Focsan, Ovidiu Oniga, Mircea Bogdan, Laurian Vlase, Ilioara Oniga, Gabriel Marc

**Affiliations:** 1 National Institute for Research and Development of Isotopic and Molecular Technologies, 67-103 Donat, 400293 Cluj-Napoca, Romania; mihaela.mic@itim-cj.ro (M.M.); calin.floare@itim-cj.ro (C.G.F.); mircea.bogdan@itim-cj.ro (M.B.); 2 Nanobiophotonics and Laser Microspectroscopy Centre, Interdisciplinary Research Institute in Bio-Nano-Sciences, Babeș-Bolyai University, 400084 Cluj-Napoca, Romania; raluca.borlan@ubbcluj.ro (R.B.); monica.iosin@ubbcluj.ro (M.F.); 3 Department of Pharmaceutical Chemistry, “Iuliu Hațieganu” University of Medicine and Pharmacy, 41 Victor Babeș Street, 400012 Cluj-Napoca, Romania; ooniga@umfcluj.ro (O.O.); marc.gabriel@umfcluj.ro (G.M.); 4 Department of Pharmaceutical Technology and Biopharmaceutics, “Iuliu Hațieganu” University of Medicine and Pharmacy, 41 Victor Babeș Street, 400012 Cluj-Napoca, Romania; laurian.vlase@umfcluj.ro; 5 Department of Pharmacognosy, “Iuliu Hațieganu” University of Medicine and Pharmacy, 12 Ion Creangă Street, 400010 Cluj-Napoca, Romania; ioniga@umfcluj.ro

**Keywords:** polyphenol, thiazole, antioxidant activity, synthesis, HSA, ITC, NMR, drug binding, molecular docking

## Abstract

Polyphenols have attained pronounced attention due to their ability to provide numerous health benefits and prevent several chronic diseases. In this study, we designed, synthesized and analyzed a water-soluble molecule presenting a good antioxidant activity, namely catechol hydrazinyl-thiazole (CHT). This molecule contains 3′,4′-dihydroxyphenyl and 2-hydrazinyl-4-methyl-thiazole moieties linked through a hydrazone group with very good antioxidant activity in the in vitro evaluations performed. A preliminary validation of the CHT developing hypothesis was performed evaluating in silico the bond dissociation enthalpy (BDE) of the phenol O-H bonds, compared to our previous findings in the compounds previously reported by our group. In this paper, we report the binding mechanism of CHT to human serum albumin (HSA) using biophysical methods in combination with computational studies. ITC experiments reveal that the dominant forces in the binding mechanism are involved in the hydrogen bond or van der Waals interactions and that the binding was an enthalpy-driven process. NMR relaxation measurements were applied to study the CHT–protein interaction by changing the drug concentration in the solution. A molecular docking study added an additional insight to the experimental ITC and NMR analysis regarding the binding conformation of CHT to HSA.

## 1. Introduction

Oxidative stress is viewed as an imbalance between the production of reactive oxygen species and their elimination by protective mechanisms, which can lead to chronic inflammation. Oxidative stress can activate a variety of transcription factors, which lead to the differential expression of some genes involved in inflammatory pathways [1]. The inflammation triggered by oxidative stress is the cause of many chronic diseases. Polyphenols have been proposed to be useful as adjuvant therapy for their potential anti-inflammatory effect, associated with antioxidant activity, and the inhibition of enzymes involved.

Phenolic compounds are responsible for major organoleptic characteristics of plant-derived foods and beverages (color and taste) [2]. Much attention has been paid to these compounds, mainly due to some of their biological properties, given that the major function of these compounds is their antioxidant ability. Indeed, they have been known to induce antimutagenic and anticancerogenic effects, but many studies have also demonstrated their harmful effects especially when applied in high concentrations (e.g., inhibition of digestive enzymes, decrease in body weight gain and growth retardation) [3]. The distribution of drugs and dietary phenolic compounds in the systemic circulation depends on, among other factors, unspecific/specific reversible binding to plasma proteins such as HSA.

Human serum albumin is the most abundant serum protein in the body and has the ability to bind a wide variety of molecules, including drugs and metabolites. Additionally, there is a wide variety of drugs that are delivered to their targeting organs or tissues by binding with HSA. Therefore, it plays a dominant role in drug disposition, affecting the biodistribution, metabolism and elimination of the molecules to which it binds [4,5]. Thus, characterization of ligand–protein interactions is vital in order to determine the pharmacokinetic profiles of these compounds and to assess their therapeutic potential.

In the first part of our present study, we developed a new synthetic molecule catechol hydrazinyl-thiazole (CHT). Its structure consists of elements strictly necessary for a good antioxidant and antiradical activity, correlated with our group’s previous findings, based on previous literature reports in the field.

In the current research, we proposed to develop a new compound with strong antioxidant activity, based on molecular hybridization, based on combining the most convenient fragments in terms of activity from previous papers reported in the field [6,7,8,9,10], with the resulted compound to have a low bond dissociation enthalpy (BDE) of the phenol O-H bonds.

No bulky or inert moieties were desired to be found in the structure of CHT and its obtention route was oriented to give a final compound as hydrochloric salt that has a good solubility in water.

In the second part of our research, we aimed to examine the mechanism of interaction between HSA and CHT. To understand this process, we used modern, specific and complementary techniques such as isothermal titration calorimetry, selective proton spin-lattice relaxation NMR experiments and molecular docking simulation methods. The above biophysical techniques are highly utilized in the field to characterize the protein–ligand interactions. Molecular docking is an important computational technique used to understand theoretical interactions of compounds with a macromolecular structure.

## 2. Materials and Methods

The reagents and solvents used for all activities presented in the presented paper (chemical synthesis, purification, spectral analysis and antioxidant assays) were purchased from local suppliers. For ITC and NMR measurements, we used HSA from Sigma-Aldrich Chemie GmbH (Schnelldorf Deutschland, Germany) and aqueous solutions were prepared in doubly distilled water and deuterium oxide, respectively.

The design of the proposed CHT compound begun from the literature observations that were confirmed by our previous research results, that catechol compounds exhibit stronger antioxidant activity than other types of diphenolic compounds such as resorcinol or hydroquinone derivatives [11,12].

Literature reports in the field of phenolic compounds indicate that the main antiradical mechanism of this class of compounds is by hydrogen atom transfer (HAT). The antiradical activity of the phenolic compounds correlates well with the easiness of the hydrogen atom’s release from the phenol groups, which in turn correlates with the bond dissociation enthalpy (BDE) [13,14,15,16].

In our previous report using in silico techniques, we have identified in the resorcinol hydrazinyl-thiazoles for the *ortho* phenol BDE ranging from 65.9 to 70.2 kcal/mol, whereas for the *para* phenol we identified BDE ranging from 80.8 to 81.1 kcal/mol [17]. Applying the same computational protocol, for the new CHT compound from the current study, the BDE for the *meta* phenol was computed to be 69.9 kcal/mol, whereas the BDE for the *para* phenol was computed to be 77.2 kcal/mol. Comparing the BDE of the phenol groups in CHT with the BDE from the compounds previously reported by our group, we can conclude that the proposed compound CHT could exhibit significant antioxidant activity in the in vitro assays.

### 2.1. Chemical Synthesis of CHT

Synthesis of intermediate compound **3** was performed using an adaptation of previously reported similar protocols and the characterization of the compound corresponds with the expected data [8,18,19].

**Figure 1:**
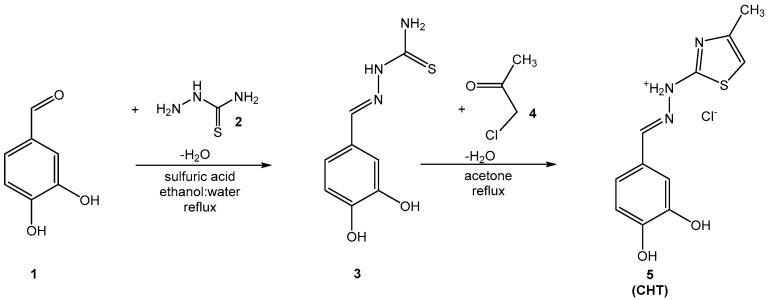
The synthesis route followed to obtain the final compound **5** (CHT).

Synthesis of compound **5** (CHT) was performed using a previously reported protocol [8,17]. Briefly, in a round-bottom glass flask, 5 mmol (1.05 g) of compound **3** were dissolved in 30 mL of boiling anhydrous acetone and 5.05 mmol (0.467 g) of chloropropanone (compound **4**) were added. After one hour the completion of the reaction was confirmed by TLC. The reaction mixture was left to cool until reaching the room temperature. The abundant precipitate was collected by vacuum filtration and crystallized twice from acetone (2 × 50 mL), to get the pure final compound **5** (CHT). The reaction path followed is presented in Figure 1.

The NMR experiments were carried out on a Bruker AVANCE III spectrometer (Bruker, Karlsruhe, Germany) to liquid state, operating at 500.13 MHz for ^1^H and 125 MHz for ^13^C, with a broad-band observation probe (BBO). All NMR measurements were recorded in a D_2_O solution at 298 K. The infrared spectra of the compounds were recorded using a FT/IR 6100 spectrometer (Jasco, Cremella, Italy) in anhydrous KBr pellets. The mass spectra were recorded on an Agilent 1100 device coupled with an Agilent Ion Trap SL mass spectrometer working in negative ionization mode.

### 2.2. In Vitro Antioxidant, Antiradical and Chelation Assays

The solid powders of CHT and reference compounds were dissolved in DMSO to give 2 mM starting sample solutions. In assays where supplementary dilutions of the starting solutions were needed, it was presented in each assay protocol.

The absorbance of the samples was determined using an UV-VIS Jasco V-530 spectrophotometer (Jasco International Co., Tokyo, Japan) at room temperature and with a spectral resolution of 1 nm. For the ^1^O_2_ scavenging assay we have used quartz glass cuvettes with an optical path of 2 mm (Hellma, Müllheim, Germany), whereas for all the other spectrophotometric determinations we used single-use 10 mm width cuvettes made from poly(methyl methacrylate) or polystyrene (Labbox Labware, Barcelona, Spain).

To avoid possible interferences the absorption spectra of CHT was recorded, indicating the lack of absorption maxima near the wavelengths at which the spectrophotometry experiments were performed to evaluate the antioxidant potential of these compounds.

All the spectrophotometric determinations were performed in triplicate and the results were presented as averages.

The CHT antioxidant capacity evaluation was performed based on the different possible mechanisms reported in the literature—antiradical, electron transfer or by the chelation of transition metals.

#### 2.2.1. Antiradical Assays

ABTS^•+^ radical scavenging assay was performed using a modified protocol, previously reported by our group [8,17]. Briefly, 10 µL, 15 µL, 20 µL, 30 µL, 40 µL, 70 µL and 100 µL of 1 mM stock solution of CHT and trolox were mixed in cuvettes with the proper amount of DMSO to get 100 µL solutions in each cuvette. Later, 3900 µL of reagent solution prepared in potassium phosphate buffer (0.1 M, pH = 7.4) and activated with MnO_2_ were added into each cuvette and mixed well. The decrease in the absorbance of the resulted solutions was determined spectrophotometrically at λ = 734 nm, against a blank sample.

DPPH^•^ radical scavenging assay was performed using a protocol previously reported by our group [8,17].

For the ^1^O_2_ scavenging assay, 1,3-diphenylisobenzofuran (DPBF) was purchased from Alfa Aesar (Haverhill, MA, USA) and Indocyanine Green (ICG), also known as Cardiogreen, was purchased from Sigma–Aldrich (St. Louis, MO, USA), and both were used without further purification. To study the antioxidant properties of CHT, we assessed its efficiency to annihilate the singlet oxygen generated by ICG molecules, a photosensitive agent, under 80 s irradiation with a 785 nm continuous-wave laser diode (Micro Raman System R-3000, 190 mW power). For this purpose, DPBF (0.1 mM) was used as an indirect chemical probe to characterize the release of singlet oxygen in solution and to calculate the singlet oxygen quantum yield (Φ(^1^O_2_)) of the CHT (10^−6^ M) and ICG (10^−5^ M) mixture in ethanol, relative to the Φ(^1^O_2_) of free ICG (10^−5^ M) in ethanol. In addition, we studied a mixture of ascorbic acid (10^−6^ M) and ICG (10^−5^ M) as standard for the antioxidant properties of CHT. Using the same experimental conditions, free DPBF in ethanol was used as a control [20]. The absorbance maxima of DPBF at 419 nm was recorded and the Φ(^1^O_2_) was calculated using the following Equation (1):

(1) $$\Phi \left({}^{1}O_{2}\right)=\Phi {\left({}^{1}O_{2}\right)}^{\mathrm{ICG}}\times \frac{S\times F^{\mathrm{ICG}}}{S^{\mathrm{ICG}}\times F}$$

where:

- Φ(^1^O_2_) is the oxygen singlet quantum yield of the investigated sample,
- Φ(^1^O_2_)^ICG^ is the ^1^O_2_ quantum yield of ICG [20],
- S is the slope of the absorbance difference of DPBF as a function of time,
- F is the absorption correction factor determined as: F = 1 − 10^−OD^, where OD is the optical density at 785 nm, the excitation wavelength.

#### 2.2.2. Electron Transfer Assays

In order to evaluate the electron donation capacity of CHT, three assays were carried out: Ferric Reducing Antioxidant Potential (FRAP), Reducing Power (RP) and Phosphomolybdate Assay for Total Antioxidant Capacity (TAC). From the reference compounds and CHT, solutions with a concentration of 0.2 mM in DMSO were obtained by diluting the stock solutions, which were subsequently used according to the protocols that our group previously reported [7,9,17]. The absorbance of the resulted mixtures in each assay was determined spectrophotometrically against a blank sample.

#### 2.2.3. Metal Ions Chelation Assays

The chelation potential of CHT for the ferrous ions and cupric ions was determined spectrophotometrically using increasing concentrations of CHT and EDTA-Na_2_, using ferrozine as an indicator for the amount of free ferrous ions in samples and murexide as an indicator for the amount of free cupric ions in samples, according to our previously reported protocol [8,17].

### 2.3. Isothermal Titration Calorimetry

To understand the mechanism of HSA–CHT binding, ITC experiments were per-formed on a Nano ITC microcalorimeter (TA Instruments, New Castle, DE, USA) at 25 °C. All the solutions were degassed under vacuum for 30 min. The HSA solution (75 μM) was maintained in the sample cell while the syringe (250 μL) was filled with the solution CHT (1 mM). The titration experiment consisted of 25 injections of 10 μL of aliquots, added intermittently every 300 s interval, which was sufficiently long for the signal to return to the baseline and to ensure the equilibrium for the system. To ensure a complete mixing of the solutions the stirring speed was kept at 250 rpm. Blank experiments were recorded under identical experimental and instrumental setup.

By integrating the raw data peaks using NanoAnalyze software (TA Instruments, New Castle, DE, USA) we obtained a plot of observed enthalpy changes per mole of injectant (kJ mol^−1^) against molar ratio. Based on an independent binding model, the corrected calorimetric data were analyzed to determine the values of the association constant (Ka), binding stoichiometry (n) and also the enthalpy and entropy changes (ΔH and ΔS) of the reaction. Using Equation (2), we determined the free-energy change (ΔG):

(2) ΔG=ΔH−T×ΔS

### 2.4. Nuclear Magnetic Resonance (NMR)

For ^1^H NMR experiments, we used an excitation pulse of 10.1 μs and a pulse sequence *zgpr* in order to suppress the water signal. For 5000 Hz spectral domain which corresponds to 65 K data points, we accumulated 64 scans. For proton decoupled ^13^C NMR spectra we used an excitation pulse of 8 μs with a spectral domain of 28,846 Hz which corresponds to 65 K data points, and 4096 scans were accumulated. The ^13^APT NMR spectrum was obtained accumulating 4096 scans with a spectral domain 65 K data points over a 28,846 Hz. The 2D ^1^H-^1^H COSY NMR spectrum was obtained using a matrix of 2048/1024 data points over 5000 Hz and 24 scans. The 2D ^1^H-^13^C HSQC NMR spectrum was obtained using a matrix of 2048 (^1^H)/2048 (^13^C) data points covering a spectral width of 5000 Hz (^1^H)/25,154 Hz (^13^C) with 16 scans.

As an alternative to ITC experiments, to put in evidence the interaction between CHT and HSA, we used the NMR relaxometry. In order to assess the CHT binding to HSA, we measured the selective spin-lattice relaxation time of ligand protons. The method used to obtain the spin-lattice relaxation times was the inversion recovery method (180°–τ–90º) with selective 180°-Gauss 1_180i_1000 soft pulse. The selective soft perturbation pulse (width: 11.2 ms and power: 51 dB) corresponds to an excitation with a frequency band of 45 Hz and 15 values for τ between 0.1–15 s were used. The exponential regression analysis of the longitudinal magnetization components was used for fitting the relaxation times, following Equation (3):

(3) A(t)=A(0){1−2exp(−τ/T1)}

### 2.5. Molecular Docking

**Figure 2:**
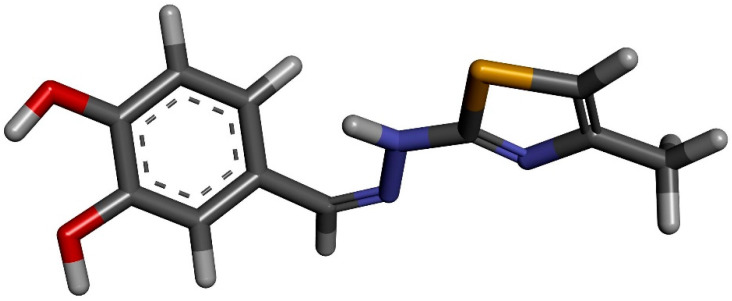
CHT optimized molecular structure using Gaussian 09.

Molecular docking of CHT molecule to HSA was performed using AutoDock v4.2 [21]. As a preliminary step, an optimization of the ligand molecular structure is necessary. We performed it using Gaussian09 software [22] with M06-2X functional and 6-311++G (d,p) basis set. This optimized structure of CHT is represented in Figure 2.

Albumin conformation was extracted from the crystallographic structure available in the Protein Data Bank PDB ID: 1AO6 [23], keeping only the chain A and removing the crystallographic water molecules.

We followed the standard docking methodology and we used the same parameters described in our previous contributions [8,24]. We do not repeat them in detail here, we will only specify the essential ones. The protein was kept rigid during the docking procedure and the ligand flexibility was taken into account by setting up 5 torsion angles around the rotatable bonds. The cubic search box, centered and surrounding the entire protein, had a grid size of 126 × 126 × 126 points with 0.750 Å grid point spacing. All hydrogen atoms were added to both albumin and CHT and the non-polar hydrogens were automatically merged during the preparation stage performed using AutoDockTools v 1.5.6 [21,25]. The Gasteiger–Marsili method [26] has been used to compute the atomic charges. The search for the best conformers has been performed using the Lamarckian genetic algorithm with the default parameters. With the aid of Biovia Discovery Studio Visualizer v20.1 and Chimera v1.14 [27] we visualized and analyzed the docking results.

## 3. Results and Discussion

### 3.1. Chemical Synthesis

Compound **5** (CHT) resulted from the Hantzsch cyclization of the thiosemicarbazone of 3’,4’-dihydroxybenzaldehyde (**3**). The synthesis route of CHT is presented in Figure 1.

The intermediate compound **3** was dissolved in boiling acetone, but the final product **5** (CHT), being a hydrochloride salt, precipitates from solvent. The analysis of the spectral data (IR, MS and NMR) recorded for the intermediate compound **3** and for the final compound **5** indicates the successful obtention of the pure compounds. For the two compounds the IR and MS spectrograms are presented in Figures S1–S4, respectively,

(E)-2-(3,4-dihydroxybenzylidene)hydrazine-1-carbothioamide (**3**): yellow-beige crystalline solid; carbonization over 240 °C (lit. 245 °C [19]); yield = 63%; FT IR (KBr) ν_max_ cm^−1^: 3468, 3447, 3330, 3179, 1621, 1604, 1593, 1540, 1509, 1366, 1330, 1281, 1169, 1118, 842, 804, 761, 619; MS: *m*/*z* = 210.5 (M − 1);

(E)-2-(3,4-dihydroxybenzylidene)-1-(4-methylthiazol-2-yl)hydrazin-1-ium chloride (CHT) (**5**): yellow solid; carbonization over 230 °C; yield = 53%; FT IR (KBr) ν_max_ cm^−1^: 3551, 3474, 3411, 3286, 3226, 3140, 3120, 2921, 1626, 1616, 1606, 1589, 1526, 1511, 1437, 1349, 1299, 1273, 1194, 1163, 1097, 965, 960, 755, 734; MS: *m*/*z* = 247.9 (M − 1); ^1^H-NMR (D_2_O, 500 MHz) δ: 2.08 (d, 3H, -CH_3_ (H6), J = 1.2 Hz), 6.32 (d, 1H, Th-C5-H (H5), J = 1.2 Hz), 6.71 (d, 1H, Ar (H3), J = 8.2 Hz), 9.92 (dd, 1H, Ar (H1), J = 8.2 Hz and 1.9 Hz), 7.00 (s, 1H, Ar (H2), J = 1.9 Hz), 7.71 (s, 1H, =CH- (H4)); ^13^C-NMR (D_2_O, 125 MHz) δ: 12.61 (-CH_3_, C11), 102.77 (Th, C9), 113.02 (Ar, C5), 115.32 (Ar, C2), 121.90 (Ar, C1), 124.88 (Ar, C6), 136.91 (Ar, C3 and C4 overlapping signals), 143.94 (Th, C8), 147.03 (Th, C10), 149.01 (=CH-, C7).

### 3.2. In Vitro Antioxidant, Antiradical and Chelation Assays

In order to get a complete image about the antioxidant potential of CHT, multiple assays were performed. Using in vitro methods we assessed the radical scavenging (ABTS^•+^, DPPH^•^ and ^1^O_2_), the electron transfer (FRAP, RP and TAC) and, complementarily, the chelation potential of metal ions (Fe^2+^ and Cu^2+^), important due to their involvement in radical generation.

#### 3.2.1. Antiradical Assays

The antiradical activity of CHT was determined spectrophotometrically as capacity of scavenging ABTS^•+^, DPPH^•^ and ^1^O_2_, compared to reference compounds ascorbic acid and/or trolox. The activity of the tested compound CHT against ABTS^•+^ is presented in Table 1. It can be seen that the activity of CHT in scavenging of ABTS^•+^ is 3.16 times more intense than of trolox.

The activity of the tested compound CHT against DDPH^•^ is presented in Table 2. It can be seen that CHT has a much stronger scavenging activity than the reference antioxidants used in the DDPH^•^ scavenging assay. CHT has a 3.28-fold lower IC_50_ than trolox and a 4.94-fold lower IC_50_ than ascorbic acid, respectively, by comparing the resulted IC_50_.

**Figure 3:**
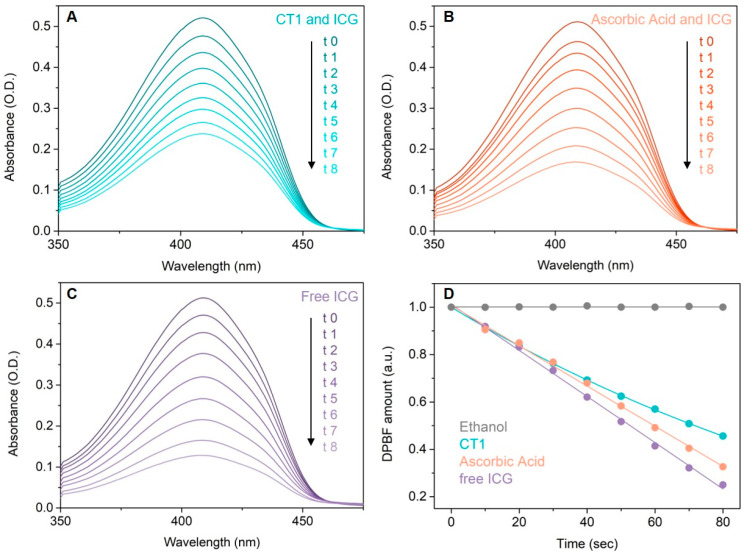
Absorption spectra of DPBF and (**A**) CHT and ICG, (**B**) ascorbic acid and ICG and (**C**) free ICG mixtures during irradiation at 785 nm, at different time points: 0, 10, 20, 30, 40, 50, 60, 70 and 80 s. (**D**) Degradation of DPBF molecules as a function of irradiation time, in the presence of CHT and ICG (blue), ascorbic acid and ICG (orange), free ICG (purple) and the control (gray).

^1^O_2_ scavenging assay was performed by evaluation of the time-dependent degradation of DPBF molecules. The presence of ^1^O_2_ in samples was quantified by the degradation of DPBF at room temperature by measuring UV-vis absorption spectra (Figure 3) at the beginning, before irradiation, and after every 10 s of irradiation for a total of 80 s. No significant modifications were observed in the absorbance spectra of DPBF during or after irradiation of the control sample (Figure 3D, gray). As expected, the biggest decrease in absorbance at 419 nm, corresponding with the decomposition of DPBF, was found in samples containing only ICG (Figure 3C), due to singlet oxygen generation (singlet oxygen quantum yield = 0.15 [20]). When ICG was associated with CHT or ascorbic acid as reference compound, a lower decrease in the absorbance was found at 419 nm, explained by the fact that DPBF is protected against the action of singlet oxygen radicals by the associated compounds (CHT or ascorbic acid). Comparing the magnitude of absorbance decrease in the absorbance spectra of DPBF when using the CHT + ICG vs. ascorbic acid + ICG, it can be seen that CHT better protects DPBF from radical degradation than ascorbic acid (Figure 3A vs. Figure 3B). Singlet oxygen quantum yield for the two samples CHT + ICG vs. ascorbic acid + ICG was found to be 0.11 vs. 0.13, respectively.

#### 3.2.2. Electron Transfer Assays

Several assays have been applied to evaluate the potency of CHT to donating electrons, as each of these experiments uses different experimental conditions, providing a clearer picture of the activity of the evaluated compound: FRAP, RP and TAC. FRAP and RP are based on the reduction of Fe^3+^ to Fe^2+^—the first assay in acidic pH (3.6), at room temperature and in presence of 2,4,6-tris(2-pyridyl)-*s*-triazine (TPTZ) as ligand for the resulted ferrous ions, is given a strong blue complex, whereas RP is performed in phosphate buffer (0.2 M, pH = 6.6) at 50 °C, resulting in Perl’s Prussian blue. In the FRAP assay, the oxidant Fe^3+^ is found free in solution, whereas in the RP assay, it is found complexed with six cyanide ions. The TAC assay is based on the reduction of Mo^6+^ to Mo^5+^, resulting in the formation of a green complex with phosphate ions.

The results of the antioxidant activity evaluation of CHT in terms of donating electrons are presented in Table 3.

The capacity of reduction of ferrous ions to ferric ions by CHT is significant, compared to ascorbic acid and trolox. In this context, analyzing numerically the data resulting from the FRAP and RP assays, shows the importance of performing as many antioxidant tests as possible in the evaluation of a compound in order to obtain information about its activity. In the case of the FRAP assay, at equimolecular concentrations CHT has a double activity compared to ascorbic acid and 1.71 times higher than that of trolox, in the case of the RP test the order is reversed—CHT having an activity 1.38 times higher than ascorbic acid and 2.53 times higher than trolox. This inversion of the order of activity between the two reference compounds can very well be explained due to the large differences in the experimental conditions under which the two assays are performed, as presented above: pH, temperature and ligands bound to the ferric oxidant. However, both the FRAP and RP assays indicated a strong activity in the reduction of the ferric ions by CHT, higher than ascorbic acid and trolox.

Analyzing the results of the Phosphomolybdate Assay for Total Antioxidant Capacity (TAC), CHT exhibited a similar activity to ascorbic acid and 80.10% of the activity of trolox.

#### 3.2.3. Metal Ions Chelation Assays

Using the ferrozine method, the chelation capacity of ferrous ions of CHT was evaluated in comparison with EDTA-Na_2_. The decrease in absorbance of samples in the presence of a complexant indicated that a lower quantity of ferrous ions were free in solution and a lower quantity of ferrozine–ferrous ions complexes resulted. The results of the ferrous ions chelation assay are presented in Table 4. Although CHT has a catechol structure, the chelating activity of ferrous ions is insignificant.

CHT exhibited a significant potential in chelation of cupric ions, as presented in Table 5. In the interval of concentrations tested, CHT presented between 81.12% of activity of EDTA-Na2 at the lowest concentration (66.66 µM) and 70.12% of activity of EDTA-Na_2_ at the highest concentration (199.98 µM). The result is not surprising, because the hydrazinyl-thiazoles are known for their complexation properties of some transitional metals [28,29,30,31].

### 3.3. Thermodynamics of Binding Interaction

**Figure 4:**
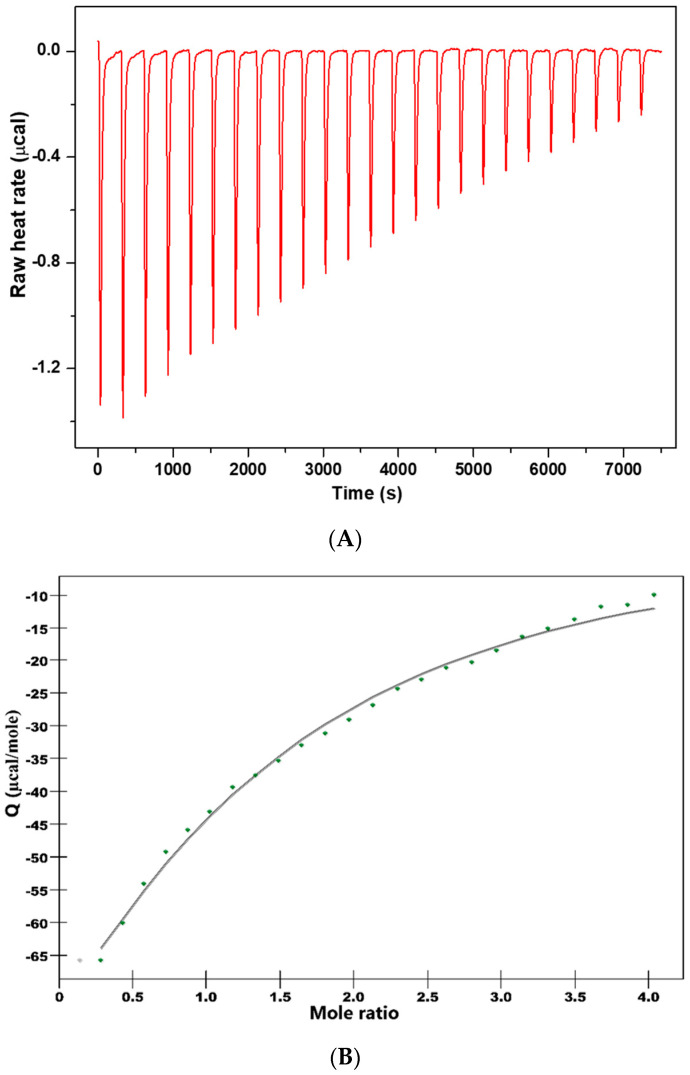
Isothermal titration calorimetric profile for the binding of CHT to human serum albumin at 25 °C. The panel (**A**) represents raw data obtained from 25 injections of 10 μL aliquots of CHT solution (1 mM) into HSA (75 µM) solution. The panel (**B**) show the incremental heat/mol of added ligand as a function of molar [CHT]/[HSA] ratio of reaction partners. By fitting the experimental data by an independent model, a solid line is obtained which represents the best-fit result.

In order to get thermodynamic binding parameters (K_a_, n, ΔH and ΔS), we performed ITC experiments. Figure 4 shows the calorimetric titration profile upon the binding of CHT to protein. Table 6 presents the thermodynamic parameters obtained by fitting experimental data with an independent binding model.

Attention was focused on the acting forces to interpret the binding mode (Figure 4 and Table 6). Analysis of thermodynamic data indicates a negative heat change which means the binding is exothermic with an affinity constant K_a_ = 7.153 × 10^3^ M^−1^. The binding stoichiometry of the process, n, was found to be 1.156.

The negative value of the Gibbs free energy (ΔG = −5.25 kcal/mol) suggest that the binding is a spontaneous process. The binding process had a favorable enthalpy (ΔH = 18.6 kcal/mol). The Gibbs energy value is associated with non-covalent interactions, protein–ligand and hydration effects as well as conformational changes which occur during this interaction.

The entropic effect of the binding process is ΔS = −44.8 cal/molK and brings an additional contribution to the negative Gibbs free energy. The values of ΔH and ΔS are informative for the nature of the forces governing the binding process. Both ΔH and ΔS are negative and the absolute value of ΔH highly exceeded the entropy terms TΔS.

Therefore, it suggests that hydrogen bonds or van der Waals interaction are involved in the binding mechanism. The thermodynamic signature of CHT and HSA interaction indicated that the binding is an enthalpy-driven process.

### 3.4. Nuclear Magnetic Resonance (NMR)

#### 3.4.1. Determination of the Molecular Structure by NMR

For a good accuracy and to confirm the proposed molecular structure of CHT, we performed the following types of NMR experiments: 1D (^1^H, ^13^C and ^13^C APT) and 2D (^1^H-^1^H COSY, ^1^H-^13^C HSQC).

**Figure 5:**
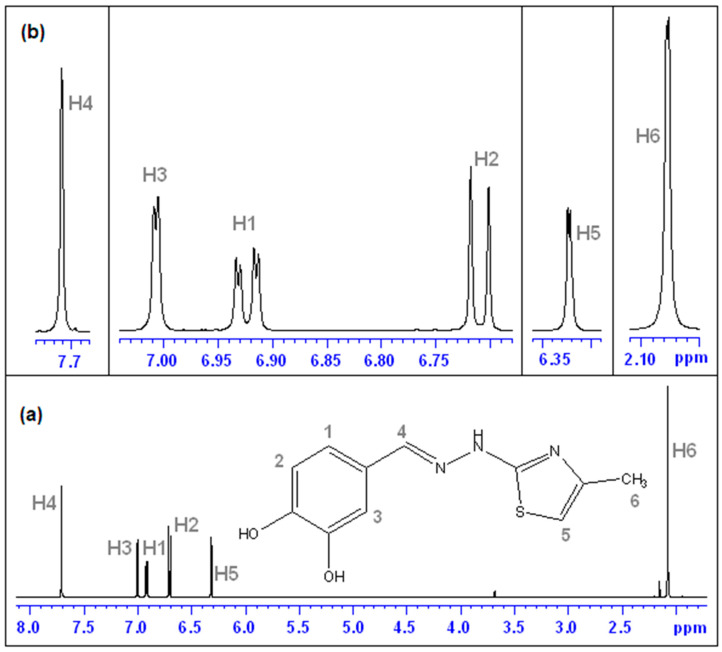
^1^H NMR for CHT in D_2_O using the *zgpr* pulse program water suppression: (**a**) total spectrum and proton numbering; (**b**) inset-details zoom.

The ^1^H NMR spectrum of CHT is shown in Figure 5. The *zgpr* pulse program was used to suppress the water signal. The assignments were conducted considering the proton numbering of the signals. In our case, we used deuterium oxide (D_2_O) as a solvent.

In this spectrum, the aromatic ring protons (H1—doublet of doublets, H2—doublets and H3—doublets) gives signals in the 6–7 ppm range. The singlet of 7.71 ppm is assigned to the H4 proton. The methyl group (H6—three protons) was found at 2.08 ppm and the H5 proton of thiazole was found at 6.32 ppm. The values of peak integrals nicely reproduce the number of protons in each group.

**Figure 6:**
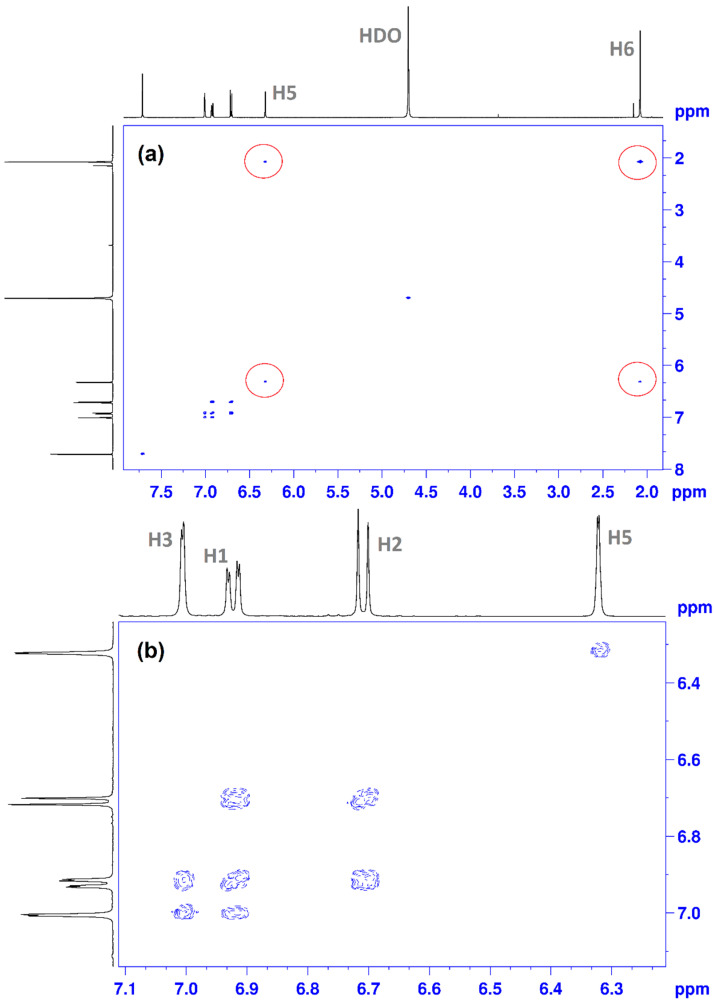
^1^H-^1^H COSY NMR spectrum of CHT in D_2_O: (**a**) total spectrum; (**b**) details zoom to aromatic area, using the proton numbering from Figure 5.

For a correct interpretation of the ^1^H NMR spectrum, we performed the 2D ^1^H-^1^H COSY NMR spectrum (Figure 6) [32]. Figure 6a presents the interactions between H6 protons of the methyl group with the proton of thiazole–H5. Interactions between other neighboring protons from the aromatic zone (6–7 ppm) in the CHT molecule are presented in Figure 6b. Thus, we have highlighted the following interactions: H3 with H1; H1 with H2.

**Figure 7:**
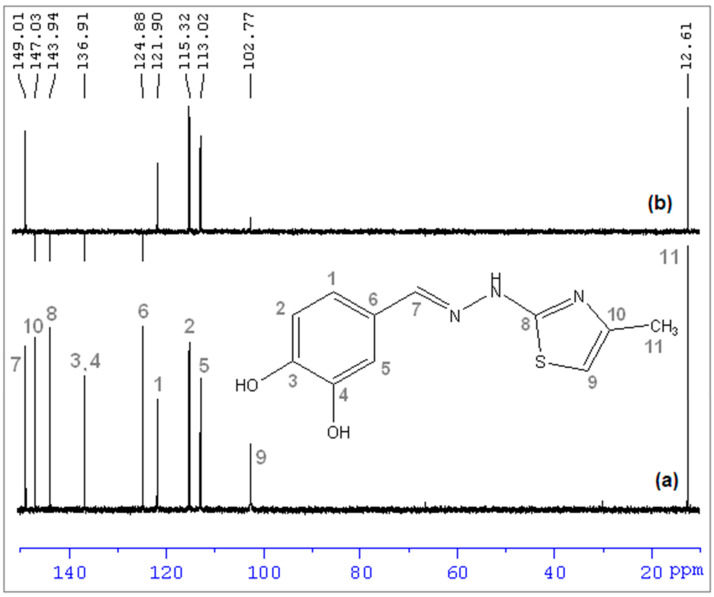
(**a**) ^13^C NMR spectra of CHT and carbon numbering; (**b**) ^13^C-APT NMR, in D_2_O.

The assignment of ^13^C NMR chemical shifts of CHT molecule is presented in Figure 7a. To identify all carbon atoms of the CHT molecule, we performed a ^13^C-APT NMR measurement (Figure 7b). This type of analysis shows all carbons including the quaternary carbon atoms, difficult to trace using traditional techniques. In the category of “up peaks” falls CH and CH_3_ carbons, whereas in the “down peaks” category would be identified carbons without any protons attached or with an even number of protons (quaternary and other similar types, such as carbons carrying phenol groups).

**Figure 8:**
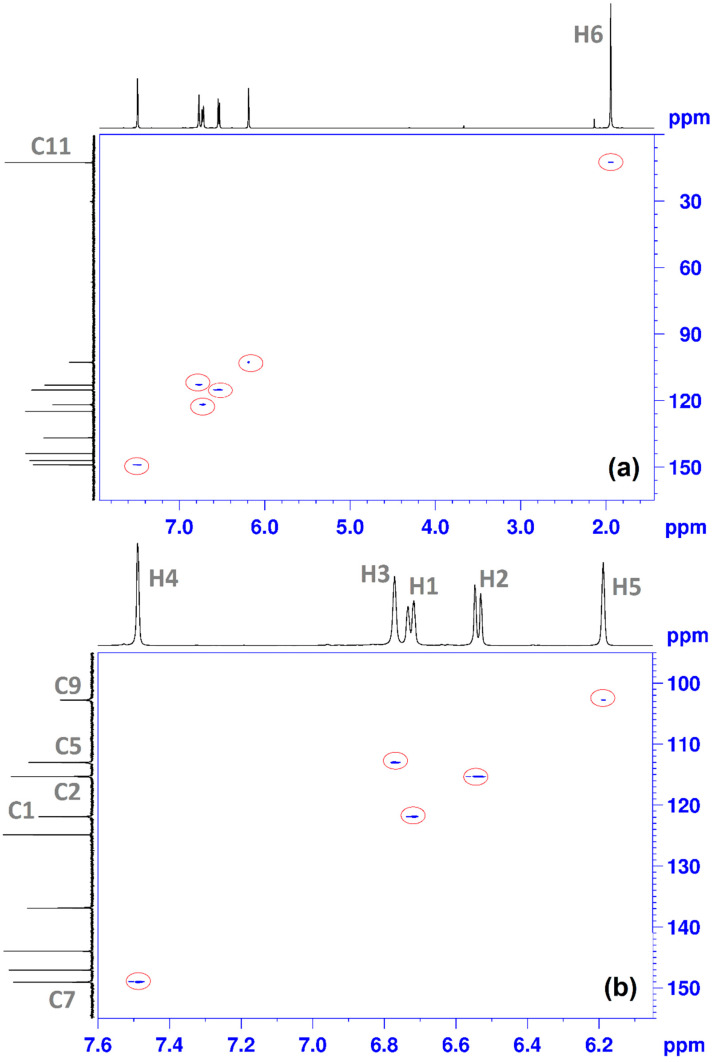
^1^H-^13^C HSQC NMR spectrum of CHT in D_2_O: (**a**) total spectrum; (**b**) details zoom to aromatic area, using the proton numbering from Figure 5 and carbon numbering from Figure 7.

^1^H-^13^C HSQC NMR was used to connect ^1^H NMR and ^13^C NMR spectra. This type of technique is often encountered in the study of the molecular structure of organic molecules [32]. The 2D NMR spectrum shows several correlations between: C11 with H6 (Figure 8a); C9 with H5; C5 with H3; C2 with H2; C1with H1; C7 with H4 (Figure 8b).

#### 3.4.2. Binding Association by NMR

To put in evidence the interaction between CHT–HSA and to determine the association constant K_a_, we used a set of samples where the concentration of the HSA was fixed to 0.2 mM and the concentration of CHT was varied from 8 to 40 mM.

For the protons H1, H2 and H4 of CHT (see proton numbering of the ^1^H NMR spectrum—Figure 5), the selective relaxation times in the presence of HAS was smaller than their corresponding values in pure D_2_O (T_1,free_(H1) = 1.613 s; T_1,free_(H2) = 2.303 s and T_1,free_(H4) = 1.791 s).

In order to obtain the binding strengths of CHT to protein we followed the variation of the selective spin-lattice relaxation for H1, H2 and H4 as a function CHT concentration.

To be able to follow the weak affinity interactions, where the CHT molecules can be found in free and bound states, we must to saturate the higher-affinity binding sites by using an excess of CHT. In this case we assume that all binding sites of CHT are independent with K_d_ = 1/K_a_ and at the same time that the binding process is a first order reversible. The relaxation rate was calculated following the Equation (4):

(4) $$R_{1\mathrm{obs}}=\frac{1}{T_{1}}=\frac{\left[\mathrm{PL}\right]}{C_{L}}\times R_{1\mathrm{bound}}+\left(1-\frac{\left[\mathrm{PL}\right]}{C_{L}}\right)\times R_{1\mathrm{free}}$$

with [PL], molecular concentration of the CHT/HSA complex; [CL], total concentration of the CHT; R_1bound_ and R_1free_, the selective longitudinal relaxation rates of the bound and free form, respectively [33].

For *n* independent binding sites, we used the below equations in order to describe the concentrations of the CHT and HSA.

(5) nCp=[PL]+[P]

(6) $$C_{L}=\left[L\right]+\left[\mathrm{PL}\right]$$

[P] being the concentration of the free HSA, and [L] the concentration of free CHT. Using the Equations (5) and (6) we obtain the following expression:

(7) $$\frac{R_{1\mathrm{obs}}-R_{1\mathrm{free}}}{R_{1b}-R_{1\mathrm{free}}}=\frac{C_{L}+nC_{p}+K_{d}}{2C_{L}}-\sqrt{{\left(\frac{C_{L}+nC_{p}+K_{d}}{2C_{L}}\right)}^{2}-\frac{nC_{p}}{C_{L}}}$$

**Figure 9:**
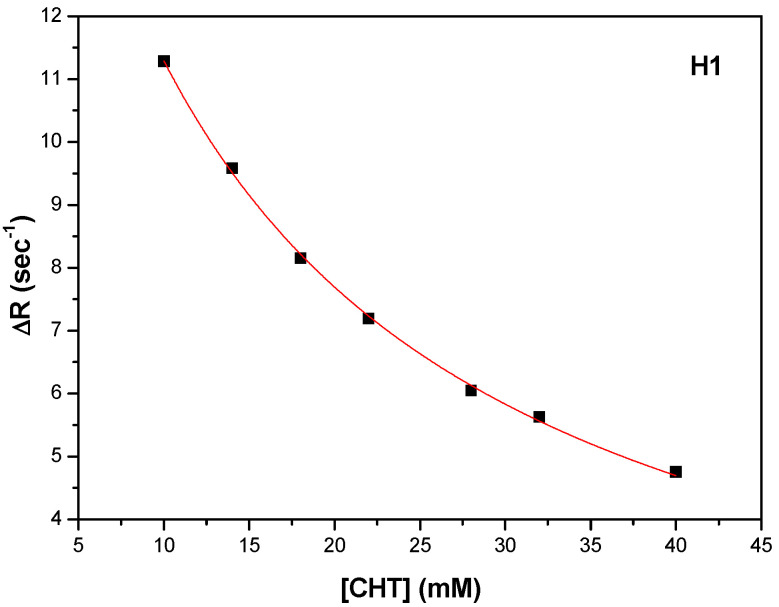
The dependence of the selective spin-lattice relaxation rate of H1 proton versus CHT concentration.

**Figure 10:**
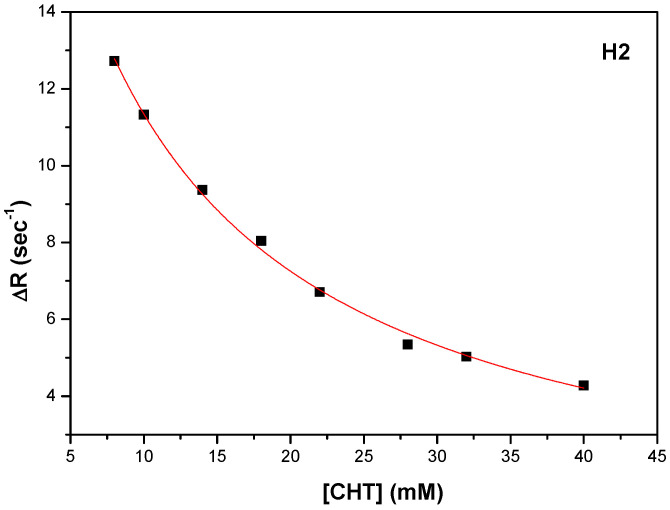
The dependence of the selective spin-lattice relaxation rate of H2 proton versus CHT concentration.

**Figure 11:**
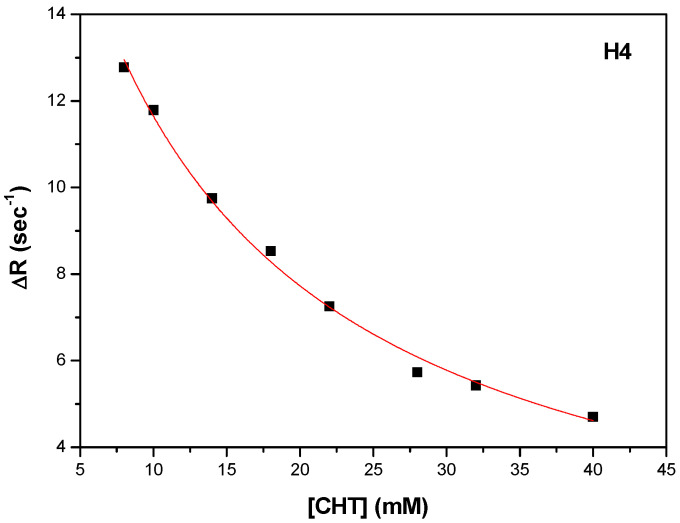
The dependence of the selective spin-lattice relaxation rate of H4 proton versus CHT concentration.

Thus, K_d_ and R_1bound_ were obtained by fitting the experimental data, represented by ΔR = R_1obs_ − R_1free_ as a function of CHT concentration. A fitting procedure, the number of binding sites was obtained n = 1, as in the ITC experiments. Figure 9, Figure 10 and Figure 11 shows the fitting procedure using Equation (7) for the H1, H2 and H4 protons of CHT, which were chosen because they present the highest variation of the spin lattice relaxation times as a function of CHT concentration:

Using the procedure described above, the results for the three protons (H1, H2 and H4) of the CHT molecule in interaction with HSA, are presented in Table 7, with an average value for K_a_ = 108.5 M^−1^. The binding affinity of the CHT evaluated by NMR method is weaker than that determined with the ITC method.

### 3.5. Molecular Docking

HSA, the most abundant protein in blood plasma, is responsible for the transport of fatty acids and a wide variety of pharmaceuticals and active metabolites, endo- and exogenous compounds. Albumin, a monomeric protein, has a predominantly α-helical secondary content and has a heart-shaped tertiary conformation.

As we also detailed in a previous study [8], the molecular structure of albumin contains three homologous domains (I-III) connected with random coils, each of them divided into two subdomains (A and B) [34]. Ghuman et al., when analyzing the drug-binding specificity of HSA, described very well the seven identified binding sites named as FA1 through FA7 where fatty acids bind to albumin [35]. Even before determining the crystallographic structure of HSA, Sudlow’s sites were proposed [36,37,38]. Sudlow’s drug site 1, DS1, is formed by an extension and widening of site FA7 towards the site FA2 and can consequently accommodate ligands bulkier than fatty acids. In a similar way, Sudlow’s drug site 2, DS2, is formed by a rearranging and merging of sites FA3 and FA4.

Albumin can then bind several types of molecules of different sizes, and even the same compound competitively, in different sites due to its intrinsic structural flexibility and many available binding sites.

**Figure 12:**
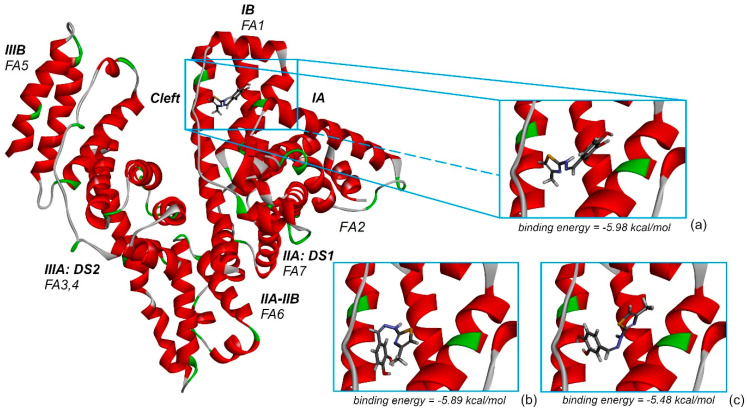
The conformation with the highest binding energy of CHT into the albumin FA1 binding pocket. FA1–FA7 of Sudlow’s drug binding sites are additionally specified. In figures (**a**–**c**) we represented a closer look at the highest binding energy conformations of the three clusters with the most numerous members which bind in the same FA1 pocket.

**Figure 13:**
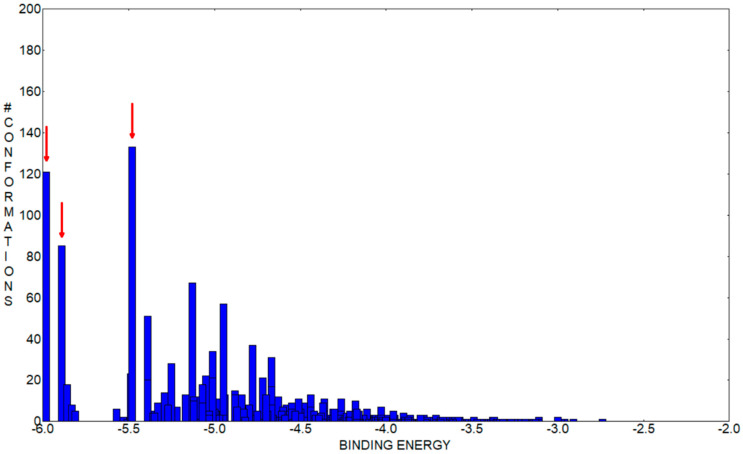
Histogram of the binding energy distribution to HSA of the cluster conformations of CHT. The three clusters having the most numerous members are specified with red arrows. Their highest binding energy conformations are represented in Figure 12a–c.

Figure 12 represents the highest binding energy conformations of CHT to HSA. It binds in the FA1 pocket of the albumin and has a value of binding energy equal to −5.98 kcal/mol. Figure 12a represents a closer look at this conformation binding site, and Figure 12b,c represents two other conformations binding in the same pocket, FA1, and which, as we will see on the following, promote this pocket to be the most favorable one to bind CHT. To better understand the analysis procedure, after the 2000 docking runs ended, the resulting conformations were organized in clusters according to their similarity. Figure 13 represents the histogram of the binding energy distribution of these docking conformations organized in clusters. The position of each blue rectangle in the histogram is determined by the value of the highest binding energy within each cluster and the intensity corresponds to the cluster number of members.

Analyzing this distribution, we initially observe that the cluster corresponding to the structure with the highest binding energy, represented in Figure 12a, has 121 members. In the histogram, we can see that this is the cluster with the second highest number of members. Additionally, the other two lower binding energy clusters, which have the most numerous amounts of members, indicated with red arrows in Figure 12, also bind in the same pocket FA1. The second highest binding energy conformation cluster has 85 members and the optimal structure has an energy of −5.89 kcal/mol, whereas the third, which is the most numerous cluster with 133 members, has the optimal structure with a binding energy of −5.48 kcal/mol. In Figure 12b,c, mainly with comparative purpose, we represented their highest binding energy conformations. In our opinion, this particular preference is a good indication that FA1 is the most favorable pocket to bind CHT. With caution, we mention here, that the other clusters within the binding energy interval [−5.98, −5.48] kcal/mol can indicate another competitively preferred binding sites. Some of these conformations bind to the FA2 and FA5 pockets but their corresponding clusters have a lower number of members and the binding energy is lower. We mentioned this particularly because, in our docking analysis, we kept rigid the guest, HSA protein, to its crystallographically determined molecular structure and the experimental presence of water molecules and possibly ions is not taken into account in the docking procedure, but all these influences can have an impact on the effective binding conformation between CHT and HSA.

**Figure 14:**
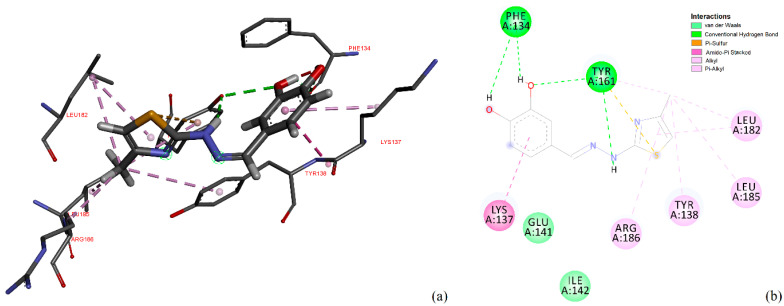
Close interactions of CHT in the albumin binding pocket (**a**) and 2D interaction map (**b**).

With these in mind, on the following, we focused our attention on the conformation having the highest binding energy in the FA1 pocket of albumin. Figure 14 represents the close interaction of CHT with the protein amino acids in the FA1 pocket.

**Figure 15:**
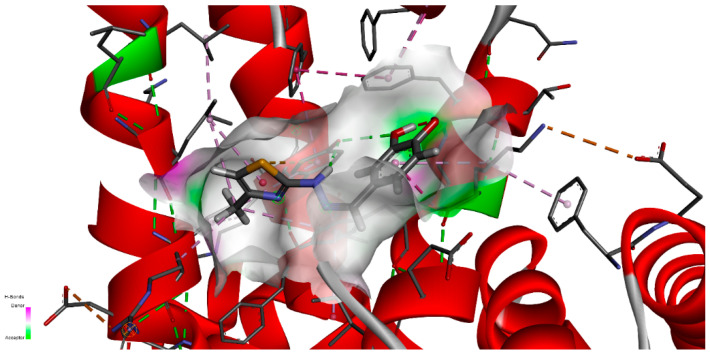
CHT in the FA1 binding pocket of the albumin including the receptor hydrogen-bond surface map.

It can be seen that CHT is stabilized in the FA1 binding site principally through hydrogen bonds of both hydroxyls of catechol with phenylalanine PHE134 and tyrosine TYR161, and their benzene rings additionally define the pocket as their Pi electrons also interactwith thiazole sulfur atoms and with the catechol ring. The binding pocket is also defined further, on the catechol side of the molecule, through the interactions with LYS137, TYR138 and with GLU141 (not shown in Figure 14a). The carboxyl of GLU141 amino acid is at about 4.5 Å from CHT H1, H2 and H4 which proved to have the most pronounced variation in selective spin-lattice relaxation in the 1H-NMR measurements. We can ascertain this interaction in Figure 15 where we also included the receptor hydrogen-bond surface map.

The conformation represented in Figure 14 and Figure 15 is the one with the highest binding energy obtained after 2000 AutoDock runs and the clustering indicated clearly, in our opinion, the FA1 pocket to be the most favorable one to bind CHT. Even if we are confident that this structure has a good chance of being the effective one, we understand that the particular conformation in liquid state, which we investigated experimentally, is probably mainly favored as a result of the interaction also with the solvent molecules. Additionally, the protein is flexible and surely there will be a small adjustment of the conformation of the amino acids in the binding pocket following the interaction with CHT.

## 4. Conclusions

Based on our previous reports in developing new phenolic hydrazinyl-thiazoles with antioxidant activity, we have presented in the current paper the obtention, characterization and evaluation of our new compound CHT. This compound was designed after analyzing the results of our previous research, guided by the preliminary computational results which indicated that a catechol moiety would be preferred to a resorcinol moiety in a hydrazinyl-thiazole compound for a higher antioxidant activity. The in vitro evaluation of the antioxidant activity of CHT indicated it has strong activity in the applied assays. In most assays, CHT exhibited a stronger activity than the reference compounds trolox or ascorbic acid.

The spectral data recorded for the compound, such as MS, IR and NMR spectra, confirm the proposed structure and the NMR absorption signals were correctly assigned based on 2D correlation spectra (^1^H-^1^H COSY and ^1^H-^13^C HSQC). CHT exhibited significant activity in the in vitro evaluation of the antioxidant properties. In terms of the chelation of the metal ions, copper (II) chelation activity of CHT is strong, whereas chelation of Fe(II) is negligible.

In the present study, the binding of CHT with HSA was investigated using ITC calorimetry, NMR spectroscopy and molecular docking, which thoroughly extended our knowledge of the mechanism involved in the drug–HSA binding process. The thermodynamic characterization in solution revealed that the CHT is able to effectively interact with HSA and enabled the determination of the driving forces for the molecular recognition. Enthalpy/entropy compensation indicates the involvement of the significant hydrophobic interaction, whereas other forces, such as electrostatic interactions, are also present.

NMR selective relaxation experiments provided quantitative information about the relationship between affinity and structure in the CHT–HSA complex. These results concluded that CHT is bound with an aromatic ring and H4 to HSA, this method can highlight locally which part of the CHT molecule is directly involved in the interaction with HSA.

We underline that both ITC and NMR techniques were able to highlight the CHT–HSA interaction with 1:1 stoichiometry, although different concentration ranges were used.

Using molecular docking we performed a systematic computational search of the most probable binding conformation. Without very much doubt, the maximum 2000 runs allowed by the docking procedure implemented in AutoDock, favored a conformation which dock into the FA1 pocket. The FA1 binding site is rather large and the clusters with the most numerous members all binding in this pocket. The identified highest binding energy conformation proved to have the protons with the most pronounced variation in selective spin-lattice relaxation at about 4.5 Å of the carboxyl of GLU141 amino acid.

## Figures and Tables

**Table 1:** Determined activity of CHT against ABTS^•+^.

Compound	Scavenging Effect (%) at Specific Concentration (µM)							IC_50_ (µM)
	2.5	3.75	5	7.5	10	17.5	25	
CHT	28.93	41.32	49.70	70.72	87.20	+	+	5.03
Trolox	−	13.10	20.45	25.99	31.72	53.26	77.62	15.90

− represents <5%, + represents >95%.

**Table 2:** Determined activity of CHT against DPPH^•^.

Compound	Scavenging Effect (%) at Specific Concentration (µM)										IC_50_ (µM)
	5	7.5	10	12.5	20	30	50	60	70	80	
CHT	28.40	37.22	48.79	57.48	81.57	+	+	+	+	+	10.80
Trolox	8.33	13.10	14.67	23.22	31.77	39.67	68.38	83.38	+	+	35.50
Ascorbic acid	−	−	9.75	12.40	20.04	30.05	46.18	56.35	66.41	73.05	53.39

− represents <5%, + represents >95%.

**Table 3:** Results of the electron transfer assays FRAP, RP and TAC, expressed as % activity of CHT compared to ascorbic acid and trolox.

Reference	FRAP	RP	TAC
Ascorbic acid	201.55	138.54	106.46
Trolox	171.35	253.86	80.10

**Table 4:** Results of the ferrous ions chelation capacity evaluation of CHT (%).

Compound	58 nM	116 nM	162.4 nM	185.6 nM	208.8 nM	232 nM	464 nM
CHT	−	−	−	−	−	−	−
EDTA-Na_2_	11.71	20.00	36.37	61.98	86.53	+	+

− represents <5%, + represents >95%.

**Table 5:** Results of the cupric ions chelation capacity evaluation of CHT (%).

Compound	66.66 µM	99.99 µM	133.32 µM	166.65 µM	199.98 µM
CHT	15.04	18.99	21.56	26.05	31.19
EDTA-Na_2_	18.54	23.89	29.21	38.01	44.48

**Table 6:** Thermodynamic parameters for HSA–CHT interaction.

Ka (10^3^/mol)	ΔH (kcal/mol)	TΔS (kcal/mol)	ΔG (kcal/mol)
7.153	−18.6	−13.36	−5.25

**Table 7:** K_d_, K_a_, R_1bound_ and *n* values, determined by NMR in the CHT–HSA interaction.

Proton	K_d_ (×10^−3^M)	K_a_ (M^−1^)	R_1bound_ (s^−1^)	n
H1	11.20	89.2	1203.5	1
H2	7.57	132.1	1002.4	
H4	9.59	104.2	1145.5	

## Data Availability

The data presented in this study are available in the article and Supplementary Materials.

## Supplementary Materials

The following supporting information can be downloaded at: https://www.mdpi.com/article/10.3390/antiox11071245/s1, Figures S1 and S2: The IR spectra recorded for compounds **3** and **5**, respectively; Figures S3 and S4: The MS spectra recorded for compounds **3** and **5**, respectively.
