# Supplementary material for: Antioxidant Activity Evaluation and Assessment of the Binding Affinity to HSA of a New Catechol Hydrazinyl-Thiazole Derivative
Source: Antioxidants (Basel). 2022 Jun 24;11(7):1245. doi: 10.3390/antiox11071245 (PMC9312188; doi:10.3390/antiox11071245)
Supplement: Supplementary file 1 [file antioxidants-11-01245-s001.zip › antioxidants-1761441-supplementary.pdf]

antioxidants

MDPI

Article

Supplementary Material

1. Figures

1.1. The IR spectra

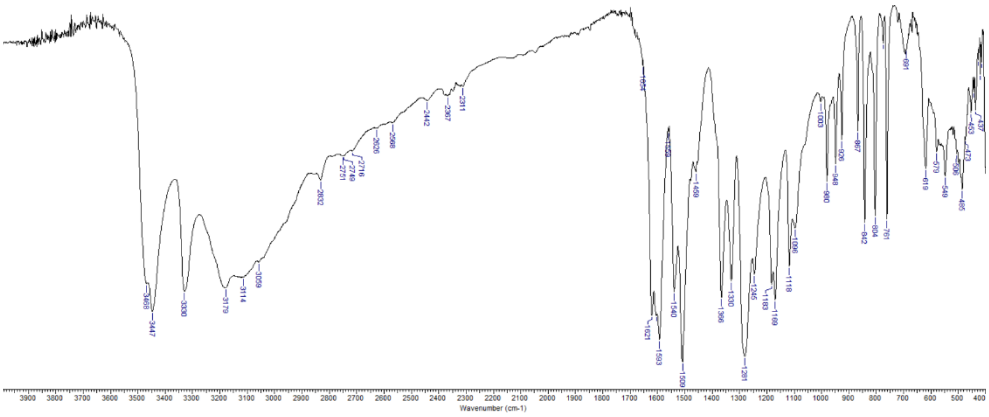

Figure S1. The IR spectrum for the compound **3.**

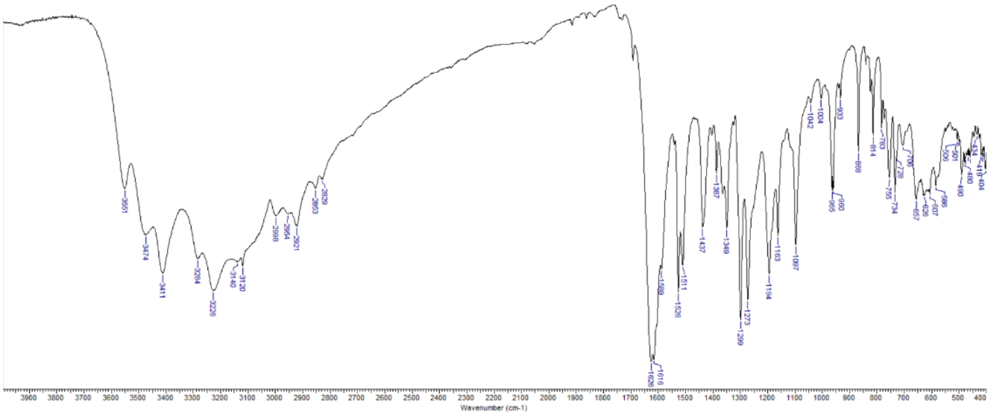

Figure S2. The IR spectrum for the compound **5.**

1.2. The MS spectra

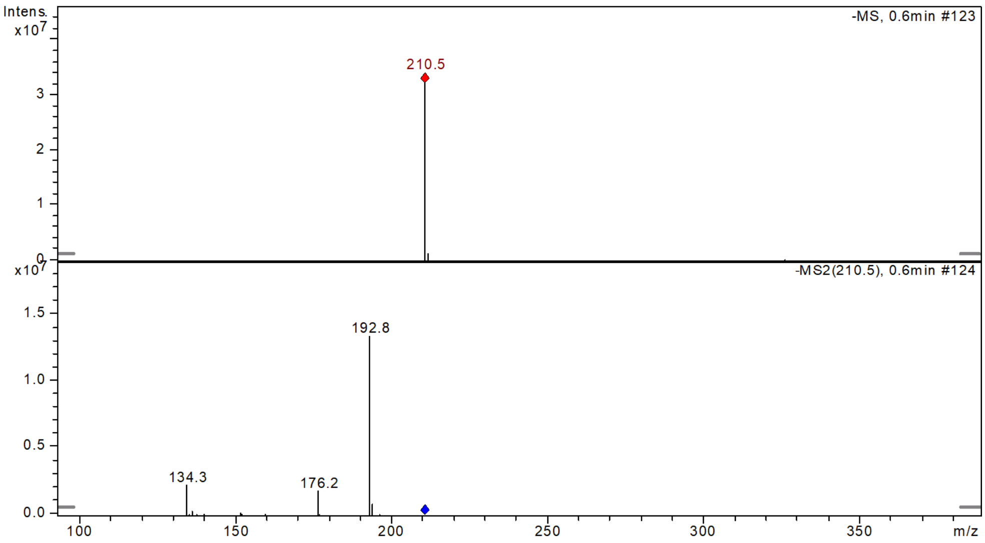

Figure S3. The MS spectrum for the compound **3.**

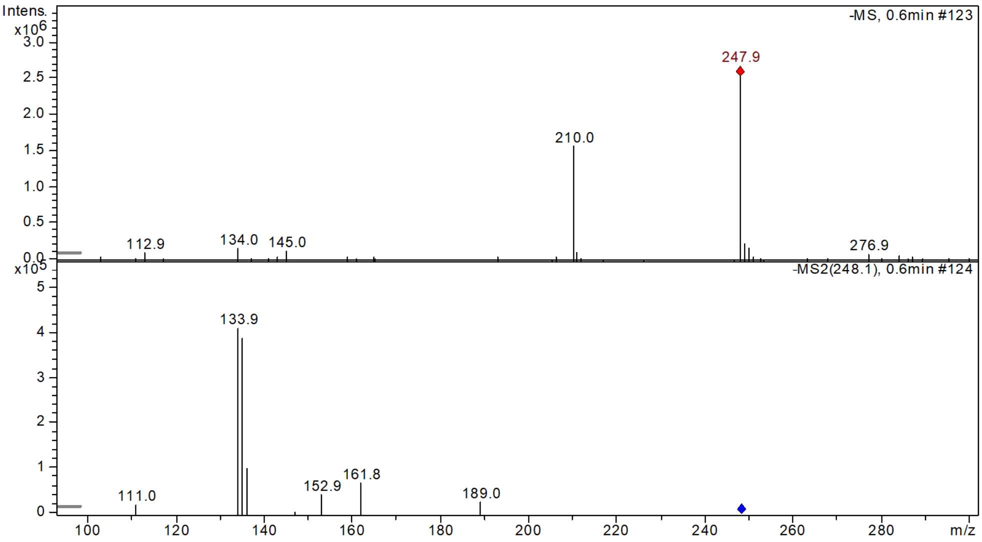

Figure S4. The MS spectrum for the compound **5.**
